# Application of WO_3_ Hierarchical Structures for the Detection of Dissolved Gases in Transformer Oil: A Mini Review

**DOI:** 10.3389/fchem.2020.00188

**Published:** 2020-04-07

**Authors:** Zhijie Wei, Lingna Xu, Shudi Peng, Qu Zhou

**Affiliations:** ^1^College of Engineering and Technology, Southwest University, Chongqing, China; ^2^Chongqing Electric Power Research Institute, State Grid Chongqing Electric Power Company, Chongqing, China

**Keywords:** WO_3_, gas sensors, hierarchical structure, oil-immersed transformer, fault characteristic gas, mechanism

## Abstract

Oil-immersed power transformers are considered to be one of the most crucial and expensive devices used in power systems. Hence, high-performance gas sensors have been extensively explored and are widely used for detecting fault characteristic gases dissolved in transformer oil which can be used to evaluate the working state of transformers and thus ensure the reliable operation of power grids. Hitherto, as a typical n-type metal-oxide semiconductor, tungsten trioxide (WO_3_) has received considerable attention due to its unique structure. Also, the requirements for high quality gas detectors were given. Based on this, considerable efforts have been made to design and fabricate more prominent WO_3_ based sensors with higher responses and more outstanding properties. Lots of research has focused on the synthesis of WO_3_ nanomaterials with different effective and controllable strategies. Meanwhile, the various morphologies of currently synthesized nanostructures from 0-D to 3-D are discussed, along with their respective beneficial characteristics. Additionally, this paper focused on the gas sensing properties and mechanisms of the WO_3_ based sensors, especially for the detection of fault characteristic gases. In all, the detailed analysis has contributed some beneficial guidance to the exploration on the surface morphology and special hierarchical structure of WO_3_ for highly sensitive detection of fault characteristic gases in oil-immersed transformers.

## Introduction

The safe and reliable operation of transformers is of vital importance for a stable and continuous power supply to the power grid (Lu et al., 2018; Zhang D. Z. et al., 2018; Zhang Q. Y. et al., 2018; Cui et al., 2019; Yang et al., 2019a,b). To date, the number of oil-immersed transformers accounts for more than 90% of the total number of power transformers, and the operating state of these power transformers will directly affect the condition of power systems (Zhou et al., 2016; Zhang X. X. et al., 2019). For a long-running transformer, partial overheating and partial discharge will lead to the decomposition of transformer oil into a variety of fault gases, namely hydrogen (H_2_), carbon monoxide (CO), carbon dioxide (CO_2_), methane (CH_4_), acetylene (C_2_H_2_), ethylene (C_2_H_4_), and ethane (C_2_H_6_) (Jin et al., 2017; Gao et al., 2019; Park et al., 2019; Wang J. X. et al., 2019). Hence, the detection of these fault characteristic gases has been extensively applied to diagnose early latent faults and evaluate the operation quality of oil-immersed transformers (Zhang et al., 2018a; Cui et al., 2019; Gui et al., 2019). In this respect, metal oxide semiconductor (MOS) gas sensors have attracted considerable attention due to their high-performance capability and wide range of applications for the detection of these fault characteristic gases in transformer oil (Zhou et al., 2013; Zhang Y. Z. et al., 2019).

Given this, various metal oxides have been investigated *via* different synthesis routes (Ge et al., 2017; Zhou et al., 2018a,b; Wei et al., 2019a). Of all the oxides, as a typical n-type metal-oxide semiconductor, WO_3_ has attracted a large amount interest due to its excellent physicochemical properties (Miao et al., 2015; Xu et al., 2019). To improve the performance of the gas sensors, sustainable efforts have been made to synthesize various nanostructures such as nanoparticles, nanorods, nanosheets, and nanoflowers (Wei et al., 2019b). Additionally, previous researchers have confirmed that these unique structures are closely related to its gas sensing properties (Yu et al., 2016). Therefore, the morphology controllable synthesis of different hierarchical WO_3_ nanostructures and the enhanced gas sensing performances thereof are of great importance to explore and discuss. In this review, we focus on the morphology controllable synthesis of hierarchical WO_3_ nanostructures including 0-dimensional (0-D), 1-dimensional (1-D), 2-dimensional (2-D), and 3-dimensional (3-D). In addition, the enhanced gas sensing performance and related mechanisms, especially the detection of the dissolved gases in transformer oil, have been introduced.

## Synthesis, Sensor Fabrication and Measurement

### Synthesis of WO_3_ Materials With Different Strategies

Up to now, various effective strategies have been proposed for preparing special surface morphologies and then fabricating WO_3_ based sensors with an enhanced gas sensing performance. Among these synthesis routes the template route, hydrothermal process, electrospinning method, and chemical deposition have all been widely used. Wang M. D. et al. (2019) synthesized three-dimensionally porous WO_3_ materials with different pore sizes via the template route, and they proposed a relationship between the pore size and the enhanced gas sensing performance. Gibot et al. (2011) reported the template synthesis of a highly specific surface area WO_3_ nanoparticle and discussed the surface properties, morphology and crystallographic structure in detail. Jin et al. (2019) developed different types of WO_3_ nanoparticles through a facile hydrothermal process and proposed the morphology controllable route of changing the proportion of the reagents. Cao and Chen (2017) used a facile CTAB (Hexadecyl trimethyl ammonium bromide)-assisted hydrothermal method to synthesize an urchin-like WO_3_ nanostructure, and a sensor based on this possessed an excellent gas sensing performance due to its special microstructure. Giancaterini et al. (2016) investigated the influence of thermal- and visible light-activation on the response of WO_3_ nanofibers via an electrospinning method. Jaroenapibal et al. (2018) presented the electrospinning synthesis of Ag-doped WO_3_ nanofibers and demonstrated an enhanced gas sensing mechanism.

### Sensor Fabrication and Measurement

**Figure 1:**
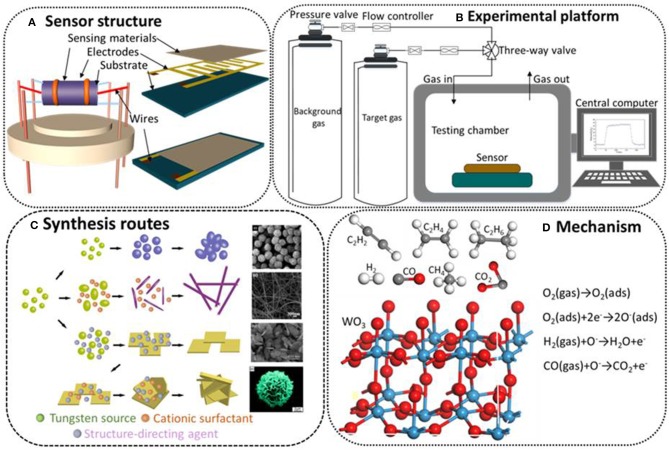
**(A)** Schematic diagram of sensor structures. **(B)** Schematic illustration of a gas sensing experimental platform. **(C)** Synthesis routes of different morphologies. Nanoparticles. Reprinted with permission from Kwon et al. Copyright (2010) American Chemical Society. Nanowires. Reprinted with permission from Wang et al. Copyright (2008) American Chemical Society. Nanosheets. Reprinted with permission from Zhang et al. Copyright (2015) American Chemical Society. Nanoflowers. Reprinted with permission from Liu et al. Copyright (2010) American Chemical Society. **(D)** Gas sensing mechanism.

To investigate the gas sensing performances of the different morphologies of WO_3_ materials, the prepared samples are used to fabricate side-heated structures, the most common versions of which are known as planar and tubular configurations. As depicted in Figure 1A, both of the structures were composed of four parts: sensing materials, wires, electrodesm, and substrate. The sensing materials in the sensor structure are prepared by dissolving the obtained WO_3_ powders into a water-ethanol mixed solution. After forming a homogeneous slurry, the paste is coated onto an alumina ceramic substrate evenly to obtain a sensing film (Zhou et al., 2019a,b). The wires are used to connect the whole measuring circuit and the electrodes are used to measure the change in sensor resistance which directly reflects the performance of the fabricated sensor (Zhou et al., 2018a). The substrate is usually made of aluminum, which can provide reliable support for sensing materials (Zhou et al., 2018c,d).

The gas sensing properties of fabricated WO_3_ based sensors are investigated using a static intelligent gas sensing analysis platform. Figure 1B presents an example gas sensor experimental process. In this set up the background gas and target gas are alternately introduced into the gas chamber to measure the characteristic dynamic response and response-recovery rate of the prepared device. The flow controller is used to adjust the flux and speed of gases in order to control their concentrations. The fabricated sensors are installed in the testing chamber and the gas sensitivity data will be directly transmitted to the central computer for processing (Wei et al., 2019c).

## Morphology Control From 0-D TO 3-D

In general, the change in sensor resistance caused by the redox reaction between oxygen molecules and test gas molecules is used to explain the basic operating principle of gas sensors. The surface morphology and special hierarchical microstructures have a crucial effect on the performance of gas sensors. In this respect, various morphologies from 0-D to 3-D with unique physical and chemical properties have been successfully synthesized and extensively explored via different effective strategies (Guo et al., 2015; Yao et al., 2015). Additionally, the controllable synthesis routes of WO_3_ nanostructures have been proposed to allow further investigation into how surface morphology affects gas sensing properties. As shown in Figure 1C, the four typical kinds of nanostructures, from 0-D to 3-D, can be controllably synthesized with different effective strategies. Given this, to further optimize the performance of WO_3_ based sensors for practical application, the exploration of surface morphology and special hierarchical structure is still a challenging but meaningful work.

### 0-Dimensional (0-D) WO_3_

As the lowest dimensional structure, 0-D WO_3_ has been investigated less as it is limited by its low specific surface area and insufficient porous structure. These disadvantages limit the diffusion and adsorption of target gas molecules during the sensing process, leading to unsatisfactory performances. Additionally, during the preparation of 0-D WO_3_ nanoparticles and the operation of the fabricated sensor, the coarsening and agglomeration of the nanoparticles might decrease the response of the device. However, various WO_3_ nanoparticles have been rationally designed and synthesized. Based on the defects mentioned above, WO_3_ nanoparticles with high dispersivity and ultra-small diameters might improve the performance of nanoparticle based sensors. In this respect, Li et al. (2019) synthesized highly dispersible WO_3_ nanoparticles with sizes ranging from 10 to 50 nm and they found the fabricated sensor exhibited an excellent gas sensing performance due to the highly effective surface area and sufficient oxygen vacancies.

### 1-Dimensional (1-D) WO_3_

1-D WO_3_ structures, for instance, nanorods, nanofibers, nanotubes, and nanowires, are considered to be beneficial nanostructures with improved special surface areas compared to. Also, the typical morphology has been applied to the detection the fault characteristic gases dissolved in transformer oil. Wisitsoorat et al. (2013) developed 1-D WO_3_ nanorods via a magnetron sputtering method, an H_2_ sensor based on which possessed prominent properties including a high response and fast response-recovery time. To further enhance the performance of 1-D WO_3_, the doping of metal ions and the introduction of surfactants have been confirmed to be effective strategies to improve the redox reaction and the orientation of special structures. Atomic platinum (Pt) is considered to be an effective doping element which can optimize the sensing properties and this strategy can be explained by the spillover effect of oxygen species and the enhancement of adsorption and desorption (Park et al., 2012).

### 2-Dimensional (2-D) WO_3_

Compared with low dimensional structures, 2-D structures possess a larger special surface area for the target gas molecules and therefore higher gas responses (Dral and ten Elshof, 2018). In comparison to the bulk 3-D structure, freestanding 2-D structures such as nanosheets, nanoplates, and thin films can provide better optimization routes including the modulation of the materials activity, surface polarization and rich oxygen vacancies. Additionally, the hierarchical microstructure assembled by rigid 2-D nanosheets possesses an open and well-defined structure which can promote the diffusion of target gas molecules (Nasir and Pumera, 2019). Especially in the field of the detection of fault characteristic gases in oil-immersed transformers, 2-D WO_3_ based sensors have been confirmed to be promising candidates with excellent gas sensing performances. Huang et al. (2020) synthesized Ru-loaded WO_3_ nanosheets via a facile impregnation method and they believed that the higher activity of surface lattice oxygens in WO_3_ nanosheets was activated by the introduction of Ru. Ou et al. (2012) fabricated H_2_ sensors based on WO_3_ nanoplates at different calcination temperatures and proved that the 2-D structure possesses a higher surface to volume ratio which clearly increased the number of surface interactive areas that could interact with H_2_ molecules.

### 3-Dimensional (3-D) WO_3_

Hierarchical 3-D structures are always assembled from diverse lower dimension fundamental blocks such nanoparticles, nanorods, and nanosheets. These various assembly routes make the hierarchical microstructures present different special morphologies, for instance, microspheres, microflowers, mesoporous structures, and other irregular structures. The well-defined structures always possess a larger special surface area and more unique microstructures, leading to better gas sensing performances including higher response times, more prominent selectivity, stability, and repeatability (Zhang et al., 2013). To detect fault characteristic gases, Zhang Y. X. et al. (2019) prepared a sea-urchin-like hexagonal WO_3_ structure created by the capping effect of potassium sulfate (which can prompt the anisotropic growth of WO_3_) and the H_2_ sensing performance was confirmed to benefit from the special hierarchical 3-D microstructure. Wei et al. (2017) synthesized hollow cauliflower-like WO_3_ by a facile hydrothermal process and found that the higher and faster response to CO might benefit from the hollow porous microstructure.

## Gas Sensing Properties and Mechanism

To improve the performances of the detection of fault characteristic gases in oil-immersed transformers, WO_3_ based sensors with different hierarchical structures have been confirmed to be promising candidates for on-line monitoring of oil-immersed power transformers due to their excellent gas sensing properties. In this section, we summarize the related works based on the recently published investigations (Table 1) and propose a plausible gas sensing mechanism.

**Table 1:** Summary of recent researches on WO_3_ based sensors for sensing of fault characteristic gases dissolved in transformer oil.

**Gas**	**Sensing material**	**Concentration**	**Temp**.	**Response**	**Refereneces**
H_2_	WO_3_ nanoparticles	200 ppm	200°C	20	Boudiba et al., 2013
	WO_3_ nanoparticles	0.5 vol%	R.T.	27.3	Xiao et al., 2018
	Pd-doped mesoporous WO_3_	5000 ppm	R.T.	11.78	Wu et al., 2019
	PdO-WO_3_ nanohybrids	40 ppm	100°C	23.5	Geng et al., 2017
	WO_3_ nanosheets	1%	250°C	80%	Rahmani et al., 2017
CO	Pt doped mesoporous WO_3_	100 ppm	125°C	10.1	Ma et al., 2018
	Cauliflower-like WO_3_	50 ppm	270°C	16.6	Wei et al., 2017
	Pt-modified WO_3_ films	20 ppm	150°C	114	Lei et al., 2016
	Pt-WO_3_ nanorods	30 ppm	300°C	4.82	Park et al., 2012
CH_4_	SnO_2_-WO_3_ nanosheets	500 ppm	90°C	1.5	Xue et al., 2019a
	Rh-modified WO_3_ films	5 ppm	350°C	63.1	Tan and Lei, 2019
	Au-WO_3_ nanowire	100 ppm	250°C	37%	Vuong et al., 2015
	SnO_2_-WO_3_ nanoplates	500 ppm	110°C	2.85	Xue et al., 2019b
C_2_H_2_	Porous WO_3_ networks	200 ppm	300°C	58	Zhang et al., 2018b
	WO_3_ nanoflowers	50 ppm	275°C	20.95	Wei et al., 2019b
	rGO-WO_3_ nanocomposite	50 ppm	150°C	15	Jiang et al., 2018

*R.T., room temperature*.

The gas sensing mechanism of the WO_3_ based sensors can be demonstrated as the change in sensor resistance caused by the redox reaction between the oxygen species (mainly O^−^) and test gas molecules on the surface of synthesized materials, as shown in Figure 1D. For typical n-type WO_3_ based sensing materials, the oxygen molecules in the testing environment will be reduced and adsorbed on the surface of the materials by capturing the electrons from the conduction band, and the target gas molecules will react with the oxygen ions and release the electrons back to the conduction band. The involved reactions can be described as follows (H_2_ and CO gas are taken as examples):

(1) $$\begin{array}{l} O_{2\left(g\right)}\to O_{2\left(ads\right)} \end{array}$$

(2) $$\begin{array}{l} O_{2\left(ads\right)}+{2e}^{-}\to {2O^{-}}_{\left(ads\right)} \end{array}$$

(3) $$\begin{array}{l} {H_{2}}_{\left(g\right)}+{O^{-}}_{\left(ads\right)}+\to {H_{2}O}_{\left(ads\right)}+e^{-} \end{array}$$

(4) $$\begin{array}{l} CO_{\left(ads\right)}+{O^{-}}_{\left(ads\right)}\to CO_{2}+e^{-} \end{array}$$

## Conclusion

In this mini review, we focus on the synthesis strategies, morphology control, sensing experimental procedures, and gas sensing performances of hierarchical WO_3_ structures from 0-D to 3-D. The gas sensing properties of various high-performance WO_3_ based sensors are summarized and discussed, especially in regards to the detection of fault characteristic gases dissolved in transformer oil. With an increasing requirement for high quality gas sensors with high responses, prominent selectivity, outstanding stability, and excellent repeatability, considerable efforts have been made to propose more effective synthesis routes, more beneficial morphology control and more accurate experiment processes. It can be foreseen that more and more hierarchical WO_3_ structures will be rationally designed and prepared due to their complicated microstructures with high special surface areas, broad internal contact area, and well-defined structures. These special hierarchical structures will provide more diffusion paths, reactive sites, and micro reaction spaces for target gas molecules adsorption, retention, and reaction. Although some achievements have been made by unremitting efforts, the further enhancement of the gas sensing properties of WO_3_ based sensors for practical applications is still a challenging but meaningful work. We hope that our work can contribute some beneficial guidance to the exploration of the surface morphology and special hierarchical structures of WO_3_. Additionally, much effort should be made to fabricate high-performance WO_3_ based sensors with predictably complicated hierarchical structures for detecting various gases, especially the fault characteristic gases dissolved in transformer oil.

## Author Contributions

All authors listed have made a substantial, direct and intellectual contribution to the work, and approved it for publication.

### Conflict of Interest

The authors declare that the research was conducted in the absence of any commercial or financial relationships that could be construed as a potential conflict of interest.
